# Google Images Search Results as a Resource in the Anatomy Laboratory: Rating of Educational Value

**DOI:** 10.2196/37730

**Published:** 2022-10-21

**Authors:** Alexandra Elisabeth Wink, Amanda N Telfer, Michael A Pascoe

**Affiliations:** 1 Division of Translational Anatomy Department of Radiology UMass Chan Medical School Worcester, MA United States; 2 Department of Cell and Developmental Biology School of Medicine University of Colorado Anschutz Medical Campus Aurora, CO United States

**Keywords:** anatomy laboratory, information literacy, internet search, anatomical images, scoring rubric, Google, images, educational value, literacy information, medical education, medical students, anatomy

## Abstract

**Background:**

Preclinical medical learners are embedded in technology-rich environments, allowing them rapid access to a large volume of information. The anatomy laboratory is an environment in which faculty can assess the development of professional skills such as information literacy in preclinical medical learners. In the anatomy laboratory, many students use Google Images searches in addition to or in place of other course materials as a resource to locate and identify anatomical structures. However, the most frequent sources as well as the educational quality of these images are unknown.

**Objective:**

This study was designed to assess the sources and educational value of Google Images search results for commonly searched anatomical structures.

**Methods:**

The top 10 Google Images search results were collected for 39 anatomical structures. Image source websites were recorded and categorized based on the purpose and target audience of the site publishing the image. Educational value was determined through assessment of relevance (is the searched structure depicted in the image?), accuracy (does the image contain errors?), and usefulness (will the image assist a learner in locating the structure on an anatomical donor?). A reliable scoring rubric was developed to assess an image’s usefulness.

**Results:**

A total of 390 images were analyzed. Most often, images were sourced from websites targeting health care professionals and health care professions students (38% of images), while Wikipedia was the most frequent single source of image results (62/390 results). Of the 390 total images, 363 (93.1%) depicted the searched structure and were therefore considered relevant. However, only 43.0% (156/363) of relevant images met the threshold to be deemed useful in identifying the searched structure in an anatomical donor. The usefulness of images did not significantly differ across source categories.

**Conclusions:**

Anatomy faculty may use these results to develop interventions for gaps in information literacy in preclinical medical learners in the context of image searches in the anatomy laboratory.

## Introduction

Information literacy is defined as the ability to recognize when information is needed, and to locate, evaluate, and effectively use needed information [1]. Regarding health information, this definition has been adapted to include the ability to “identify likely information sources and use them to retrieve relevant information, assess the quality of the information and its applicability to a specific situation, and analyze, understand, and use the information to make good health decisions” [2]. Medical students in their clerkship years and medical residents rely heavily on online resources such as UpToDate and Google for general study as well as when preparing for patient encounters [3-5]; thus, these learners need to develop strong information literacy skills [6].

Gross anatomy is often one of the first experiences of a learner’s medical school career, and therefore the anatomy laboratory is seen as an opportunity to teach and assess professional skills such as accountability and teamwork [7]. Because the anatomy laboratory is also an environment for (digital) information-seeking [8], this environment is one in which faculty can promote information literacy as a professional skill.

Many anatomy laboratories are equipped with computers or other internet-enabled devices that allow medical students to access dissection instructions and other course materials. Laboratory faculty often observe students performing web searches for images of anatomical structures [8], presumably as an alternative to using a hard-copy atlas or another course-sanctioned resource. This is consistent with medical students’ general preference for online resources such as Google and Wikipedia [3,9,10]. Potential reasons for this preference are the ease of access and interactivity associated with a search engine compared to flipping through a hard-copy textbook [11,12] or a perceived insufficiency of their other course materials [13].

Criticisms of Google Images include that the results are not specialized, detailed textual information is missing, image quality is variable, irrelevant results are time-consuming, and, importantly, images are not reliable or from valid sources [11]. The extent to which online resources are suitable for medical students has been debated by previous investigators [14-16], and this is perhaps dependent on the complexity and objectiveness of the subject matter. Few analyses of online images have been conducted, and those that were performed focused on certain medical specialties and conditions [17].

Medical students’ predilection for Google Images searches in the anatomy laboratory raises concerns about information literacy. Kingsley et al [18] found that students who preferred Google to other sources lacked the ability to retrieve and evaluate evidence-based information. Further, Google’s accessibility and ease of use may outweigh any concerns about the accuracy and trustworthiness of information [3]. In a study of online resource use by medical residents, Duran-Nelson et al [4] suggested that when using online resources, residents may value speed over quality of information. Assuming learners hold the same preferences when seeking online information in their preclinical years, a learner may select a top Google Images search result regardless of its educational quality. To address gaps in information literacy in preclinical learners, an examination of the content of these online search results is justified.

Given the gaps in knowledge presented above, the objectives of this study were to (1) report the sources of top Google Images search results for anatomical structures and concepts and (2) evaluate these images for their educational quality.

## Methods

### Ethical Considerations

No ethical approval was required for this study as there were no human subjects; thus, this did not meet the criterion of "human subjects research" as defined by federal regulations and the UMass Chan Medical School Institutional Review Board.

### Image Search Retrieval

To gather top Google Images search results for anatomical structures and concepts, one author (AEW) searched for 5-10 “high-yield” anatomical structures, groups of structures, or relations representing each of the regional content areas taught in a typical medical gross anatomy course: back and limbs, thorax, abdomen, pelvis, and head and neck. These structures included emphasized (eg, bolded) terms in laboratory manuals/dissection instructions, and structures that were frequently emphasized in didactic sessions or tested on practical examinations.

The Google Images searches were performed in January and May of 2020. Google places images closer to the top of the search results if the image is located centrally or at the top of a webpage, or if the webpage or image has been updated recently. Authority of the website is also an important factor in signaling where an image is ranked on a search results page [19]. Screenshots were taken of the top 10 image results for each term and organized in a slideshow file shared among the authors.

### Source of Images

For each image, the name of the website that published the image was recorded and the website was visited to ascertain the following information: (1) author/creator, (2) target audience, and (3) mission/purpose of the website. Two authors (AEW and MAP) created and defined the categories of websites posthoc based on one or more of these three criteria. After creating and defining the categories, the two authors (AEW and MAP) sorted the websites into these categories independently and then compared their categorizations to calculate initial percent agreement. Every disagreement in categorization was then resolved through discussion to arrive at the final categorization.

### Educational Quality

#### Relevance

An image was defined as “relevant” if it depicted the searched structure [20], and more particularly, if the image was of human anatomy (eg, an illustration of the broad ligament of the uterus of a horse was deemed not relevant). Images classified as not relevant were excluded from further analysis.

#### Accuracy of Images

All three authors independently assessed the relevant images for errors; if no errors were detected, the image was classified as “accurate.” Errors, as defined in this study, included mislabeled structures, and misrepresentations of anatomical structures, locations, and relationships. Anatomical variants, pathological presentations, omissions (eg, a structure not depicted for the sake of simplicity), minor misspellings (eg, supraspinatous vs supraspinatus), and outdated terminology no longer accepted by *Terminologia Anatomica* were not considered errors.

#### Usefulness

Usefulness of an image was defined broadly by whether an image would allow a learner to successfully locate or identify the structure in a human anatomical donor during dissection. The lack of an existing, validated rubric to assess the usefulness of anatomical images according to this definition necessitated its development in this investigation. All authors constructed this rubric following the procedures outlined by Moskal and Leydens [21] and Mertler [22]. The authors validated the initial iteration of the rubric using a small sample of images, and then modified the rubric domains and definitions to rate all of the images. The domains present on the rubric were (1) completeness, (2) cognitive load, (3) realism, (4) accuracy, (5) representation, (6) labeling of intended structure, and (7) accessibility. Definitions of these criteria and a description of each level of the rubric are found in Multimedia Appendix 1. The maximum score possible on the rubric was 28 points. To create a binary classification of useful versus not useful, we established a threshold score of 25 points. This score precludes an image receiving the lowest score of 1 point in any criterion on the rubric without receiving a score of 4 in every other criterion.

Each author rated the usefulness of the relevant images independently. Following the individual ratings, the reliability of the scoring rubric (ie, interrater reliability) was assessed using the Cronbach α calculation (SPSS version 24, IBM). The median of the three individual scores was established as the final usefulness score for each image. To determine if there was any difference in usefulness based on the source of the image, final usefulness scores (dependent variable) were compared across website categories (independent variable) using a Kruskal-Wallis test. An α level of .05 was used to determine statistical significance.

## Results

### Search Result Overview

Thirty-nine anatomical structures and concepts were identified and the top 10 image results were collected, yielding a total of 390 image results. The 390 results were sourced from 130 distinct websites. The sites that appeared in the results with the highest frequency are shown in Table 1.

**Table 1:** Most frequent sources of Google Images search results for anatomic structures.

Website title and URL	Website description/tagline	Total number of results
Wikipedia (en.wikipedia.org)	“The Free Encyclopedia”	62
Kenhub (kenhub.com)	“Learn Anatomy Faster”	21
Teach Me Anatomy (teachmeanatomy.info)	“The Ultimate Resource for Healthcare Professionals & Medical Students”	21
Pinterest (pinterest.com/ch)	“A visual discovery engine for finding ideas like recipes, home and style inspiration, and more”	18
Science Direct (sciencedirect.com)	“Elsevier’s premier platform of peer-reviewed literature”	17
Earth’s Lab (earthslab.com)	“Setting up a new place where learning becomes habit”	16
YouTube (youtube.com)	Online video-sharing and social media platform	14
Quizlet (quizlet.com)	“A free website providing learning tools for students including flashcards, study and game modes”	13
Springer Link (link.springer.com)	“Providing researchers with access to millions of scientific documents from journals, books, series, protocols, reference works and proceedings”	12
Get Body Smart (getbodysmart.com)	“A fully animated and interactive eBook about human anatomy and physiology”	10

### Source of Images

Evaluation of the websites and discussion between authors resulted in the creation of six distinct categories: (1) Health Professions Education, (2) Patient/Public Education, (3) General Reference, (4) Academic Reference/Research Articles, (5) Social Media, and (6) Other. Definitions and descriptions of each category are provided in Table 2.

Agreement between authors on categorization was strong, with 82 of the 130 distinct sites (63.1%) placed in the same category by both authors during the first independent categorization. The 48 conflicts were resolved through discussion and reexamination of the websites to arrive at the final categorization.

**Figure 1:**
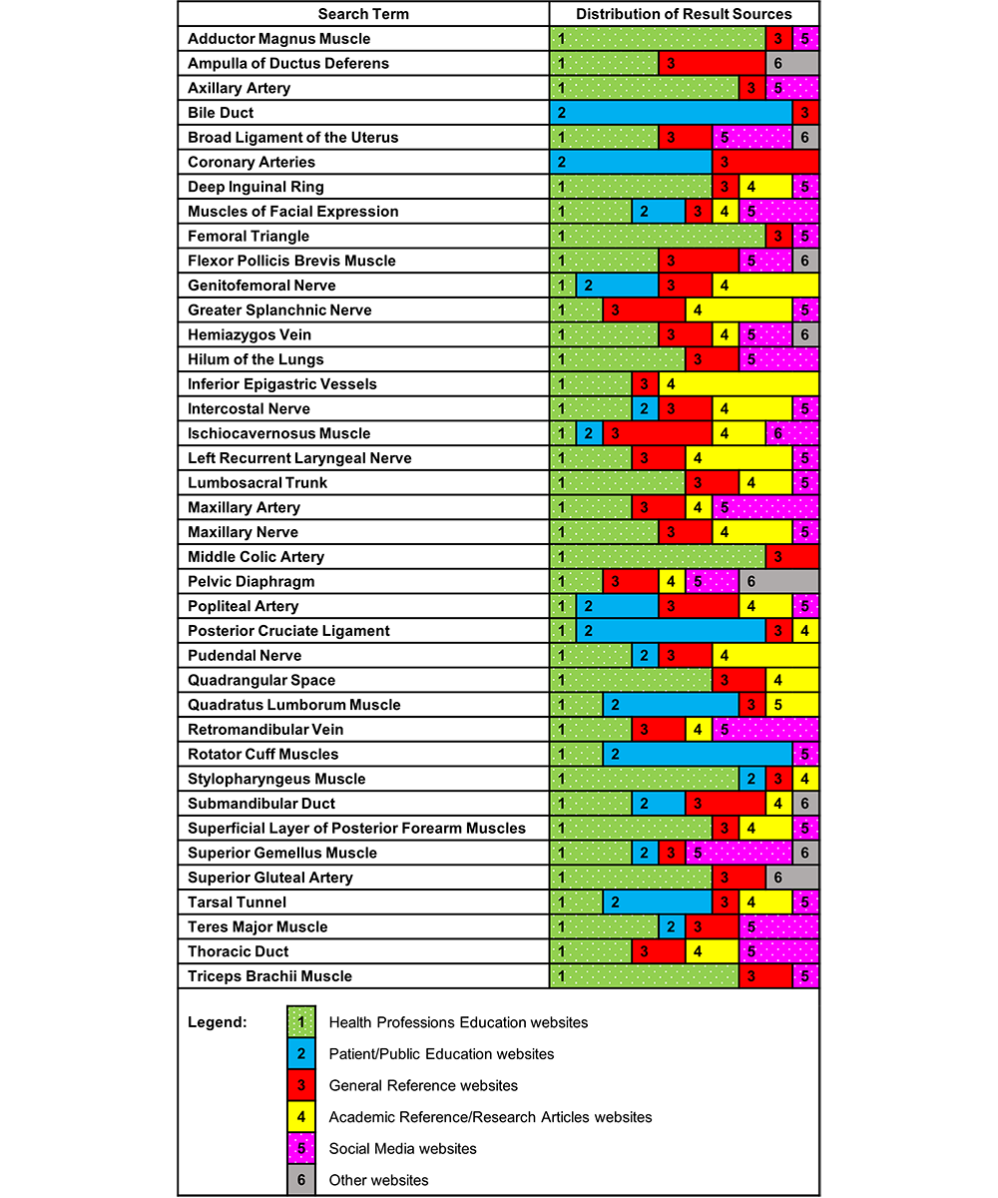
Distribution of source website categories for the top 10 Google Images search results for 39 anatomical structures (390 images).

Of the 390 image search results, 147 (37.7%) were found on Health Professions Education websites, 73 (18.7%) were found on General Reference websites, 54 (13.8%) were found on Patient/Public Education websites, 52 (13.3%) were found on Academic Reference/Research Articles websites, 50 (12.8%) were found on Social Media websites, and 14 (3.6%) were found on websites categorized as Other. The distribution of image source categories for each structure is shown in Figure 1.

Health Professions Education websites included commercial anatomy tutoring sites, as well as medical school exam study sites and specialty-specific physician resources. All but two structures (Bile Duct and Coronary Arteries) had Images search results from Health Professions Education websites.

All but one structure (Rotator Cuff Muscles) had search results from General Reference websites, although these types of websites did not comprise a majority of the search results for any of the searched structures. Wikipedia had the highest frequency of appearance of the search results (62/390 images, 15.9%). The images published on the Wikipedia entries were either public domain images (with or without modifications) from sources such as Gray’s *Anatomy of the Human Body* or images published under creative commons licenses.

Patient/Public Education websites included public-facing provider and clinic websites as well as general health information sites. Of the 39 search terms, 16 yielded results from these websites. This category also yielded the highest number (44) of distinct sites, with no site being repeated more than four times. Structures with a high frequency of results from these sites were found on pages relating to injury (eg, rotator cuff tears, back pain) or disease (eg, bile duct cancer, coronary artery disease).

Twenty-three of the 39 structures had Images search results from Academic Reference/Research Articles websites. Inferior Epigastric Vessels had the highest number of results from these sites (6/10), with five of these results coming from one book chapter. Images on these sites included depictions of variations, pathologic presentations, and surgical approaches (in which the searched structure may have been altered or removed).

Twenty-six of the 39 structures had Images search results on Social Media websites, although Social Media sites did not comprise a majority of the search results for any of the searched structures. Several images (n=14) were stills from YouTube videos; therefore, the site publishing the image was recorded as YouTube and categorized as Social Media. In these cases, the name, and where possible, a description of the account publishing the video were identified. Occasionally, images from Social Media sources were identical to images from other sources yielded by the search. This was likely due to image sharing to social media sites (eg, Pinterest) from original sources.

Only 9 of the 36 structures yielded Images search results from sites categorized as Other. These were primarily images available for purchase from stock image repositories as well as images found on sites whose primary purpose was to generate advertising revenue.

**Table 2:** Descriptions, definitions, and examples of website categories for Google Images search results.

Category name	Definition/description	Examples
1. Health Professions Education	Reference material for people working in or studying the medical sciences; assumes the audience has a baseline level of specialized technical knowledge (or is studying to acquire such knowledge) about the medical sciences	Kenhub (kenhub.com), Radiopaedia (radiopaedia.org), Statpearls (statpearls.com)
2. Patient/Public Education	Accessible health-related information for patients and the lay public, typically (but not necessarily) authored by an expert, clinician, or institution	Mayo Clinic (mayoclinic.org), WebMD (webmd.com), American Cancer Society (cancer.org)
3. General Reference	Material presented as a synthesis of several sources of information tailored to a general audience	Wikipedia (en.wikipedia.org), Exploring Nature Science Education Resources (exploringnature.org)
4. Academic Reference/Research Articles	Database of peer-reviewed articles or texts, tailored to an academic audience (includes academic publisher websites)	Science Direct (sciencedirect.com), McGraw Hill Medical (mhmedical.com), Journal of Neurosurgery (thejns.org)
5. Social Media	Platform for sharing user-generated content within a community (includes blogs)	Pinterest (pinterest.com), YouTube (youtube.com), Karmic Seeds Body Mind & Spirit (karmicseedsyogaandfitness.blogspot.com)
6. Other	Media that do not fit into one of the previous categories with no meaningful content (includes commercial stock image repositories)	Redbubble (redbubble.com), Shutterstock (shutterstock.com)

### Educational Value

#### Relevance

Of the 390 images, 25 were classified as “not relevant” because they either did not depict the searched structure (24/25, 96%) or the searched structure was depicted in a nonhuman species (1/25, 4%). Two additional images were removed from the analysis because the associated text was not in English. In total, 27 images were omitted from further analysis. The structures with the most nonrelevant images were Broad Ligament of the Uterus (4/10 results excluded), Inferior Epigastric Vessels (3/10 results excluded), and Submandibular Duct (2/10 results excluded). Of the remaining searched structures, 18 structures had one nonrelevant image and 18 structures had no excluded images.

#### Accuracy of Images

**Figure 2:**
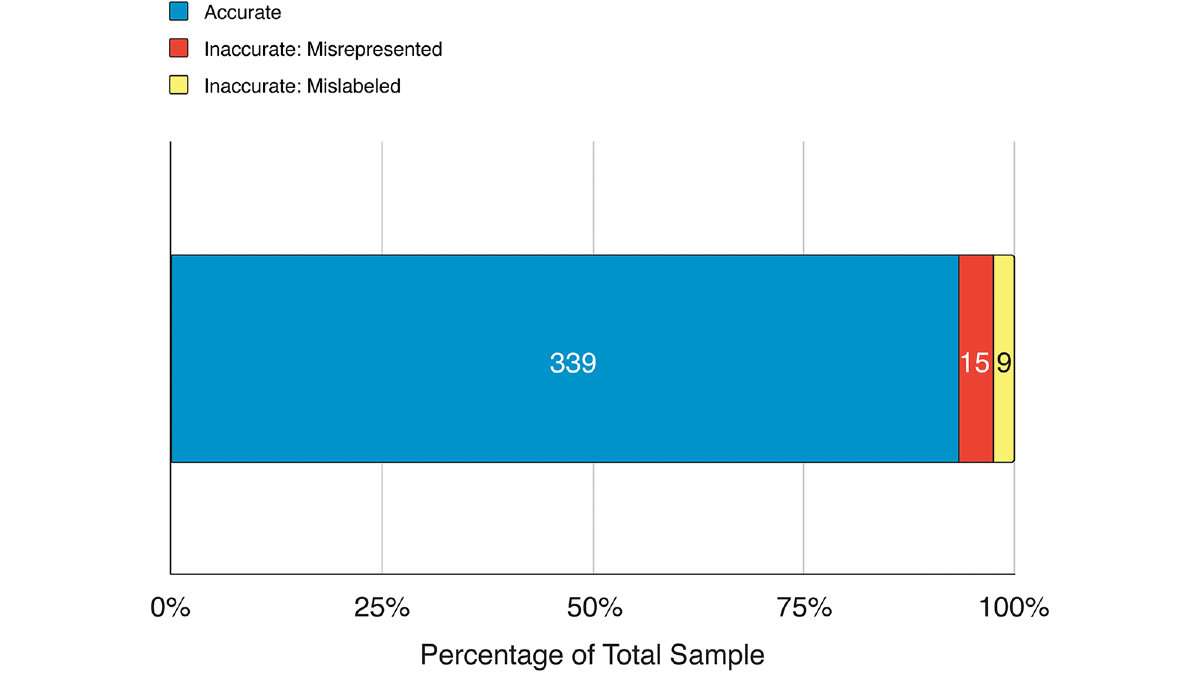
Summary of accuracy of 363 relevant images of anatomical structures. Images were determined to be accurate or containing errors such as misrepresentations of a structure’s morphology or mislabeling of a structure.

Of the 363 relevant images, 339 were accurate (93.4%) and 24 (6.6%) contained one or more errors. These errors were classified as either misrepresentations of a structure’s morphology, location, or relations (15/24, 63%), or mislabeled structures (9/24, 38%; Figure 2). Examples of errors of misrepresentation included a retromandibular vein not dividing (morphology), an intercostal bundle between the external and internal intercostal muscles (location), and a popliteal vein lateral to the popliteal artery (relation). The structures with the most images containing errors were Pelvic Diaphragm (4 images), Retromandibular Vein (3 images), Popliteal Artery (2 images), Intercostal Nerve (2 images), Quadratus Lumborum Muscle (2 images), and Middle Colic Artery (2 images). The remaining 33 structures had either one image with errors (9/33, 27%) or zero images with errors (24/33, 73%).

#### Usefulness

**Figure 3:**
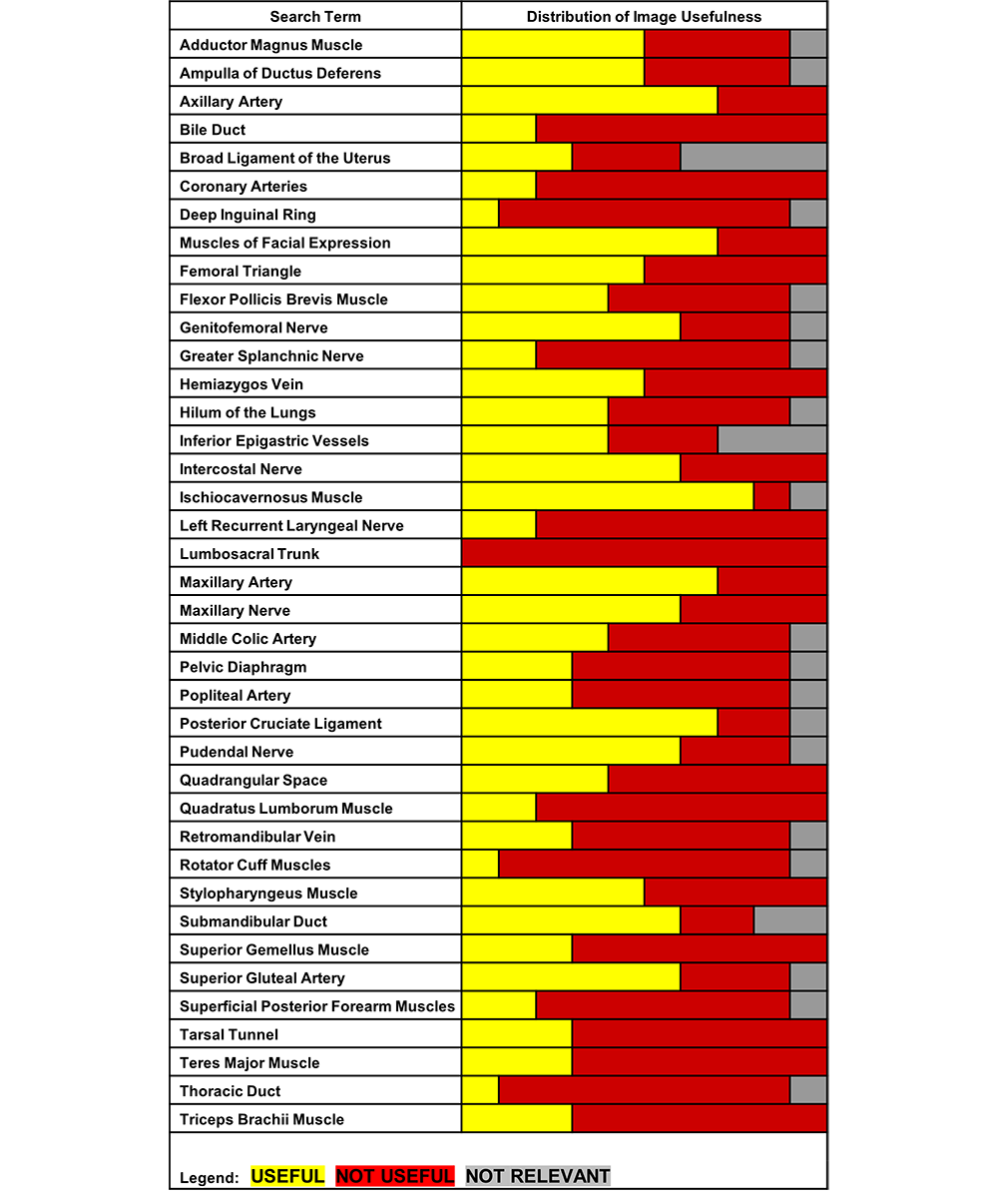
Usefulness of the top 10 Google Images search results for 39 anatomical structures (390 images).

A scoring rubric was developed to assess the usefulness of the relevant image results. Reliability among the three raters was strong when independently rating the usefulness of each image (Cronbach α=.902, 95% CI .883-.918). When the final usefulness score for each image was derived, the median usefulness score across all 363 relevant images was 24 (range 16-28). Of the 363 relevant images, 156 images (43.0%) were deemed useful when using the binary definition of a usefulness score of 25 points or greater. The structures with the most (>60% of relevant images) useful image results were Ischiocavernosus Muscle, Axillary Artery, Muscles of Facial Expression, Maxillary Artery, Posterior Cruciate Ligament, Maxillary Nerve, Intercostal Nerve, Stylopharyngeus Muscle, Genitofemoral Nerve, Pudendal Nerve, Superior Gluteal Artery, and Submandibular Duct. The only structure with zero useful images in the top 10 results was the Lumbosacral Trunk. The distribution of the number of useful images across structures is shown in Figure 3.

**Figure 4:**
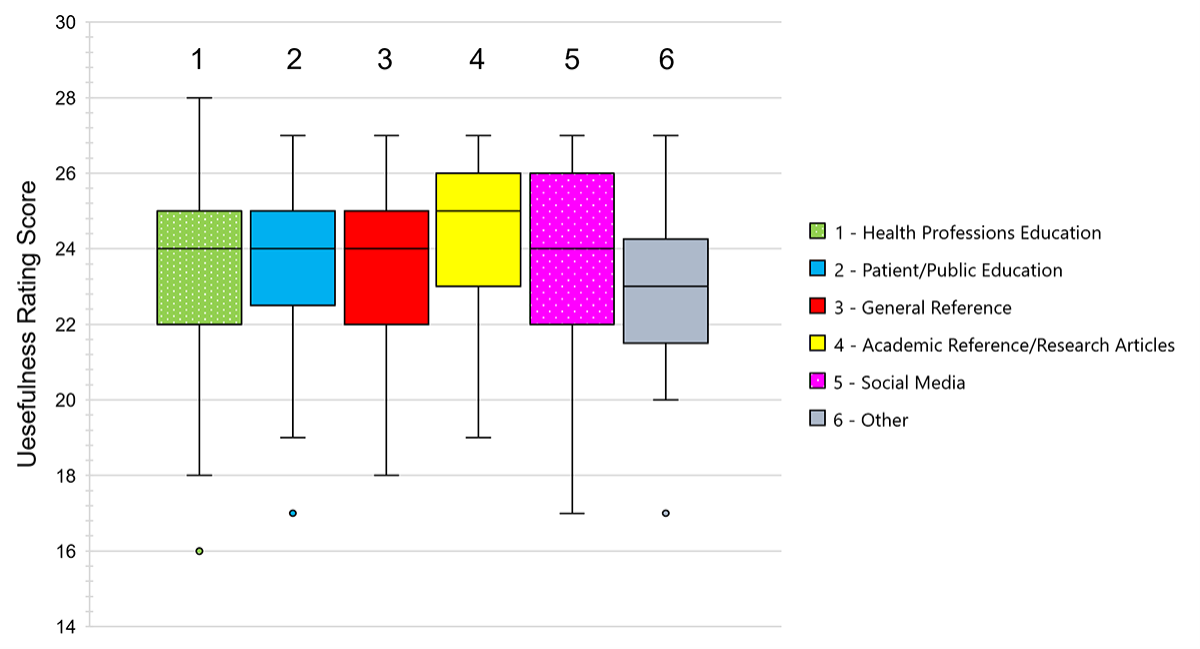
Usefulness of relevant Google Images search results for anatomical structures by source website category.

There was no statistically significant difference in median usefulness score across the six image source categories (*P*=.17; Figure 4). The percentage of useful images (score of 25 points or greater) varied across each category: 46.7% (64/137) of Health Professions Education images, 32% (17/53) of Patient/Public Education images, 45% (30/67) of General Reference images, 54% (25/46) of Academic Reference/Research Article images, 36% (18/50) of Social Media images, and 20% (2/10) of images found on Other sites were useful.

## Discussion

### Source of Images

Google Images searches were performed for 39 “high-yield” anatomical structures and concepts and the top 10 results were analyzed to determine their source and educational quality.

The largest proportion of Google Images search results were published on sites whose target audience is students and health professionals (147/390, 37.7%). These sites assume that their audience has a baseline level of knowledge of human anatomy (or is in the process of acquiring such knowledge). These sites included commercially available anatomy tutoring sites. Johnson et al [23] reported that while some learners found these sites helpful in learning anatomy, students tended to prefer materials that were specifically tailored to their courses. Not all websites in this category are necessarily held to the same standard of peer review, nor was the intention of each image to aid in locating a structure in the anatomy laboratory; therefore, images from these sites cannot be generalized as useful, as will be discussed below.

Patient and Public Education sites comprised 13.9% of the sources for the Images search results (54/390). These included general information sites (eg, WebMD, MedlinePlus), public-facing sites for major medical centers (eg, Mayo Clinic), and private provider (physician or allied health professional) websites. This category also included wellness sites that did not appear to be affiliated with any provider or practice. Although images from some of these sites can be used with confidence [17], using caution with unfamiliar sources is advised. A concern with these images is that to be accessible to the lay public, they may not provide adequate detail when applied to the study of anatomy [24]. Additionally, images published on clinical websites may represent an injured or pathological structure (eg, a torn posterior cruciate ligament) or a structure that has been surgically reconstructed and no longer resembles the typical anatomical presentation.

General Reference sites comprised 18.7% of the Images search results. Wikipedia comprised the majority of sites categorized as General Reference (62/73) and was the most frequent source of Images search results overall. Wikipedia is a popular resource among medical students [9,10,25], but its reliability is debated [26]. Arguments against Wikipedia as a resource cite poor quality of images [16] and insufficient detail [15,16,27]. London et al [14] found that Wikipedia was generally accurate and complete for basic anatomical information, despite asserting that textbooks should still be considered the gold standard. Images published on Wikipedia articles provided accurate depictions of anatomical structures; however, their educational value in terms of helping students identify and locate a structure in the laboratory varied. For instance, older public domain images published on Wikipedia (such as those from Gray’s Anatomy) included outdated terminology no longer accepted by *Terminologia Anatomica*. Because of the open-source nature of Wikipedia, it is an educational opportunity for anatomists to edit content as well as create and publish their own content [14,28].

Academic Reference sites comprised 13.3% (52/390) of the search results. These included pages that link to journal articles and other academic literature. These sites may seem attractive as reliable sources as they are peer-reviewed; however, students browsing images from these sites should be aware that this literature may include case reports of anatomical variants (such as atypical branching of the middle colic artery or congenital absence of the retromandibular vein) and may not reflect a typical anatomical presentation.

Social Media images comprised 12.8% (50/390) of the search results. The role of social media in anatomy education has been well-examined [29]. Pinterest was the fourth-most frequent source of Images search results overall, with 18 results coming from that platform. Pinterest allows users to collect and organize images and has been considered a potential source for clinical specialists to curate educational images [30,31]. Fourteen of the search results were stills from YouTube videos. YouTube is a popular educational resource, especially for learners who are considered “digital natives.” Although the usefulness of YouTube as an anatomy resource has been questioned [32], this platform presents an opportunity for anatomy educators to create and promote educationally useful content [33]. Finding an anatomy image on a social media site suggests that a learner may have found the image to be educationally beneficial and therefore worthy of sharing with others. However, for many of the anatomy images from social media platforms seen in this study, it was not immediately clear who shared the image or what the original source of the image was, which may (rightfully) lead learners to question the trustworthiness of these images.

### Educational Value

#### Relevance

An image was deemed “relevant” if it depicted the search term, although appropriate labeling was assessed separately using the usefulness rubric. This parallels the concept of “visual relevancy” as described by Sedghi et al [34], which is dependent on the learner’s ability to recognize what they are looking for in an image. Of the 390 Images search results, 363 were deemed relevant. A high proportion of relevant images in the top 10 search results for an anatomical structure reduces the effort required by learners to scroll through multiple search results to find the structure they are seeking.

#### Accuracy of Images

The accuracy of the Images search results exceeded the authors’ expectations, with 93.4% of images containing no errors. This finding may alleviate some concerns that students are being misled or taught incorrectly from online image search results. Other studies of online medical images found similar levels of accuracy [17,35], although the authors of these studies questioned the ability of nonexperts to determine accuracy [35]. This warrants further study of the ability of learners to appraise the accuracy of online anatomical images. This accuracy assessment was brought into the usefulness rubric (described below) and was one of the seven criteria used to assess usefulness.

#### Usefulness

##### Overview

The rubric for usefulness determined that 156 of the 363 relevant images (43.0%) would be useful in helping a learner locate or identify the searched structure in an anatomy laboratory setting. There was no statistically significant association between the source of the image and the usefulness of the image.

The usefulness rubric consisted of criteria supported by evidence to be of value when selecting an image that would assist a learner in locating an anatomical structure. It is important to note that what makes an image useful is highly subjective [13] and varies from structure to structure.

##### Completeness

The ability to locate an anatomical structure is dependent on an understanding of its location in the body and the key relations between surrounding structures. The criterion “completeness” was included to reflect these combined needs. An image would receive a low score if there was no sense of where in the body the structure is located and if it did not show relations to neighboring structures. The ubiquity of anatomic variation prevents the identification of anatomic structures based solely on absolute terms (eg, the occipital artery is always the third branch of the external carotid artery [36]). An example of a low-rated image (1 point) for completeness was a search result for Lumbosacral Trunk: the image was a freestanding sketch of the lumbar plexus that gave no indication of where in the body the nerve was located and showed no relations to surrounding structures.

##### Cognitive Load

According to cognitive load theory, labeling on images beyond what is relevant and a lack of focus can result in a diversion of the learner’s attention or mental activity (ie, increased extraneous load). This is particularly problematic in the anatomy laboratory setting, which is associated with a high complexity of content and skill (ie, intrinsic load). Cognitive load theory states that learning is negatively impacted if the combination of extraneous and intrinsic load exceeds the limited working memory of the learner [37]. Additionally, it has been strongly shown that multimedia full of irrelevant information distract the viewer from the main focus [38] and may impede a learner from identifying a structure in the anatomy laboratory. By rating the amount of extraneous material and the clarity of focus, the rubric captured how well an image result would assist a learner without overburdening them. For example, an image result showing the Left Recurrent Laryngeal Nerve had an excessive number of labels (45 counted) and received a low rating (1 point). In contrast, an image showing the Broad Ligament of the Uterus was rated high (4 points) because it contained all relevant labels (uterus, broad ligament, uterine tube, ovary) and the focus of the image was extremely clear.

##### Realism

The extent to which an image resembled the cadaveric presentation of the searched structure was reflected in the realism criterion. The majority of the published images were illustrations and schematic diagrams, and only two images were cadaveric photographs. Schematic diagrams are typically designed to highlight key structures through simplified or abstract representation [39]. Their usefulness in the anatomy laboratory is questionable because learners may not be able to translate these simplified images to their real-life presentations on a donor [39]. For example, a search result for Deep Inguinal Ring that depicted the ring as one end of a line-drawn cylinder representing the inguinal canal may be useful as an explanation of the structure of the inguinal canal, but would not help a learner locate the deep inguinal ring on the deep surface of the abdominal wall. In contrast, a high-quality illustration or photograph would help a learner translate the image to a cadaveric specimen more easily. The images rated in this analysis were two-dimensional images; whether images that depicted structures in three dimensions (eg, GIFs or animations that allow rotation) would improve the usefulness of an image warrants further investigation [40].

##### Representation

Learners benefit the most from viewing images of typical anatomy when they are attempting to locate structures in the body. A challenge arises for learners when viewing an atypical image as reference (eg, showing pathology, variation, or surgical reconstruction). For example, an image result showing the popliteal artery with impingement at the gastrocnemius was an atypical variation and would not assist a learner in identifying that structure in its usual location.

##### Labeling

Presenting an image of an anatomic structure to a learner with little context necessitates appropriate labeling to indicate the target structure. Despite the intuitive nature of this principle, it was recognized that some image results did not provide labeling (or other indications such as leader lines) of the searched structure; therefore, this criterion needed to be part of the rubric.

##### Accessibility

Learning materials should be inclusive and accessible to learners with a wide variety of abilities. The use of color without any other labeling to indicate structures on an anatomical image is problematic for learners with color vision deficiency [41]. Low-resolution images and images obscured by watermarks may also be visually inaccessible, or at the very least unappealing, to learners who prefer a high-quality, unobscured image [11]. An example of a low-rated (1 point) image for accessibility was a result for Maxillary Nerve that indicated the divisions of the trigeminal nerve using red and green (colors unable to be distinguished by those with protanopia, deuteranopia, and achromatopsia).

### Recommendations Based on Findings

The following recommendations are offered to educators who work with students in an anatomy laboratory setting based on the findings of our analysis. These recommendations are not necessarily universal but can be tailored to individual curricula or educational approaches.

Students have no trouble finding and accessing online resources, but they cannot necessarily discern a good resource from a bad one [23]. The students surveyed by Johnson et al [23] expressed that they need direction from educators to find reputable online sources. Nevertheless, O’Carroll et al [3] found that medical students accessed Google as a resource with high frequency despite being instructed to choose more reputable sources such as bibliographic databases. Translating this to the anatomy laboratory environment, learners will be likely to use Google Images searches despite any attestation that atlases or other course materials are the gold standard. Thus, anatomy faculty should be prepared to advise learners on best practices for Google Images searches in the anatomy laboratory.

The number of results produced by a Google Images search requires students to be aware of how to filter them effectively [6,42]. The results of the current analysis of Google Images search results for anatomical terms could be instrumental in developing guides for students on how to select reliable images for their study. These guides could include a summary of the types of websites publishing these images with guidance on how to interpret media on these sites, as well as a list of “faculty-recommended” sources.

Students should consider that websites publishing anatomical images may have agendas beyond anatomy education. These include websites promoting controversial scientific stances (eg, the Institute for Creation Research, whose mission is to promote research within the context of biblical creation) or websites advertising commercial products (eg, Whole Life Challenge, a subscription-based wellness and lifestyle brand). While the images published on these sites may be accurate and useful, there remains opportunity to assist learners in becoming aware of these agendas when selecting images from these sites.

When advising students on internet resource use in the anatomy lab, the opportunity arises to remind students of ethical behavior in the context of choosing resources. When selecting an image, students should be aware of whether the information they are using is plagiarized (eg, lecture slides shared without permission, blogs that copy text from other published material) or is published on a site that exists primarily for the purpose of helping students cheat on exams (including social media pages that circulate an institution’s previous exam questions).

### Limitations of the Study

We acknowledge that the selection of search terms is a subjective process, based on one’s own experience with anatomy curricula. Some websites were no longer active at the time of secondary analysis; either the domains had expired or the company publishing the site had ceased operations. In these cases, however, sufficient descriptive information about the website was available to properly categorize the site. It is also worth noting that the usefulness of an image may vary based on the dissection approach utilized in the course. The current analysis assumed a regional anatomical dissection approach, and images useful for this approach may not prove to be useful in more surgically based dissection protocols.

### Future Work

In the future, we intend to survey anatomy students to gauge their perceptions of anatomical Images search results and to determine whether images deemed “useful” by students meet the criteria for usefulness as defined in this rubric. These results would validate this rubric and guide the development of images in educational materials. It would also be of interest to assess learners’ ability to determine the relevance and accuracy of online anatomy images (ie, will learners be able to detect inaccuracies or a lack of relevance in an image that appears to be useful at first glance?). It would also be insightful to survey students on their overall perception of the types of websites that publish images yielded in online searches. These data would further assist educators in developing best-practice guides for anatomy image searches, such as a one-page informational handout.

### Conclusions

Medical and health professions students must develop information literacy skills for selecting appropriate resources early in their training. This skill development may take place in the anatomy laboratory as students search for online images to assist them in locating and identifying structures. A large number of Google Images search results were acquired for highly relevant anatomical structures and concepts. These images were reliably categorized, with a plurality sourced from Health Professions Education websites. Wikipedia articles appeared the most frequently among the images collected, which falls in line with the high traffic and public domain status of its images. A high percentage of images were determined to be accurate, with errors in representation of morphology, location, or relations being the most common. A scoring rubric was successfully developed and used to reveal that only 43.0% of images were useful for identifying a structure in a human anatomic donor. Usefulness scores did not differ significantly across image source categories. Taken together, these results illuminate the need for students to consider the source and quality of anatomic images that they access frequently. This presents an opportunity for the development and distribution of guidelines to assist students of anatomy.

## Appendix

Multimedia Appendix 1 Scoring rubric developed to determine usefulness of each relevant Google Images search result.
