# Supplementary material for: Google Images Search Results as a Resource in the Anatomy Laboratory: Rating of Educational Value
Source: JMIR Med Educ. 2022 Oct 21;8(4):e37730. doi: 10.2196/37730 (PMC9636525; doi:10.2196/37730)
Supplement: Multimedia Appendix 1 [file mededu_v8i4e37730_app1.docx]

|  | **Level and Description** | | | |  |
| --- | --- | --- | --- | --- | --- |
| **Criterion** | **Beginning**  **(1 point)** | **Developing**  **(2 points)** | **Accomplished**  **(3 points)** | **Exemplary**  **(4 points)** | **Criterion Score** |
| **Completeness** | No sense of where in the body the structure is located AND does not show relations to neighboring structures | Shows some neighboring structures but not body position OR shows position in the body with no neighboring structures | Proper demonstration of where the structure is located AND shows some relations to neighboring structures | Proper demonstration of where the structure is located AND shows proper relations |  |
| **Cognitive Load** | No labels at all OR so many labels it is not at all clear what the focus of the image should be | Image contains some labels AND the focus of the image is slightly clear | Image contains most relevant labels AND the focus of the image is moderately clear | Image contains all relevant labels AND the focus of the image is extremely clear |  |
| **Realism** | Schematic or line drawing with geometric shapes | Cartoon representation missing detail or with incorrect proportions or relations | Textbook/atlas style illustration with sufficient detail to resemble the real structures | Cadaveric photograph |  |
| **Accuracy** | Errors present |  |  | No errors present |  |
| **Representation** | Atypical (pathology or variation) |  |  | Typical |  |
| **Labeling of Intended Structure** | NO |  |  | YES |  |
| **Accessibility** | NO: Image is low resolution OR obscured (watermark) OR colors are problematic for color vision deficiency |  |  | YES: Image is high resolution AND color is appropriately used to clarify structure of interest |  |
|  |  |  |  | Total Score (x/28 points) |  |
